# Monocytes promote UV‐induced epidermal carcinogenesis

**DOI:** 10.1002/eji.202048841

**Published:** 2021-04-02

**Authors:** Iva Lelios, Sebastian A. Stifter, Virginia Cecconi, Ekaterina Petrova, Mirjam Lutz, Dilay Cansever, Sebastian G. Utz, Burkhard Becher, Maries van den Broek, Melanie Greter

**Affiliations:** ^1^ Institute of Experimental Immunology University of Zurich Zurich Switzerland; ^2^ Comprehensive Cancer Center Zurich Zurich Switzerland

**Keywords:** Monocytes, Langerhans cells, Cutaneous squamous cell carcinoma, Macrophages, UV radiation

## Abstract

Mononuclear phagocytes consisting of monocytes, macrophages, and DCs play a complex role in tumor development by either promoting or restricting tumor growth. Cutaneous squamous cell carcinoma (cSCC) is the second most common nonmelanoma skin cancer arising from transformed epidermal keratinocytes. While present at high numbers, the role of tumor‐infiltrating and resident myeloid cells in the formation of cSCC is largely unknown. Using transgenic mice and depleting antibodies to eliminate specific myeloid cell types in the skin, we investigated the involvement of mononuclear phagocytes in the development of UV‐induced cSCC in *K14‐HPV8‐E6* transgenic mice. Although resident Langerhans cells were enriched in the tumor, their contribution to tumor formation was negligible. Equally, dermal macrophages were dispensable for the development of cSCC. In contrast, mice lacking circulating monocytes were completely resistant to UV‐induced cSCC, indicating that monocytes promote tumor development. Collectively, these results demonstrate a critical role for classical monocytes in the initiation of skin cancer.

## Graphical abstract

Different subsets of mononuclear phagocytes accumulated in the tumor of a UV‐induced model of cutaneous squamous cell carcinoma (cSCC). While cSCC developed in the absence of Langerhans cells and dermal macrophages, mice lacking classical monocytes were completely protected from tumor formation, indicating a critical role for monocytes in tumor development.

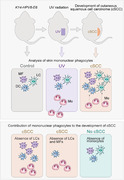

## Introduction

Exposure to sunlight is the major risk factor for the development of cSCC. UV‐induced DNA damage can trigger mutagenesis and tissue destruction, eventually leading to keratinocyte transformation and carcinogenesis. UV radiation (UVR) further contributes to cutaneous malignancies by modulating the immune microenvironment [1]. The skin comprises a network of different myeloid cells, which further expands in inflammatory conditions due to recruitment of monocytes and neutrophils. Within the healthy epidermis, the only resident myeloid cells are Langerhans cells (LCs). Similar to tissue macrophages, LCs arise predominantly from fetal liver monocytes and self‐maintain locally independent of circulating precursors in steady state [2]. However, inflammation induced by high‐dose UVR, for example, triggers the generation of monocyte‐derived LCs, which are distinct from steady‐state resident LCs [2, 3, 4]. In the context of cSCC, the role of LCs has been controversial. In a model of 7,12‐dimethylbenz(a)anthracene (DMBA)/12‐O‐tetradecanoylphorbol‐13‐acetate (TPA)‐induced cSCC, LCs enhanced mutagenesis and papilloma formation, but also inhibited tumor formation by recruiting NK cells [5, 6]. In a cSCC model induced by chronic low‐dose UVR, LCs contributed to the production of pro‐inflammatory cytokines and recruitment of innate lymphoid cells, resulting in a moderately decreased papilloma formation [7]. Thus, whether LCs promote or restrict epidermal tumor formation remains largely unresolved.

In addition to giving rise to inflammation‐induced LCs, Ly6C^hi^ monocytes also give rise to monocyte‐derived cells/macrophages. In cancer, monocyte‐derived tumor‐associated macrophages can support tumor progression by directly promoting angiogenesis or by suppressing anti‐tumor immunity. However, independent of their fate, the role of inflammatory monocytes at early stages of cancer development is not well understood.

Here, we used *K14‐HPV8‐E6 (HPV8‐E6)* transgenic mice expressing the *E6* oncogene from human papilloma virus 8 (HPV8) in keratinocytes [8] to delineate the roles of mononuclear phagocytes in UVR‐induced epidermal tumorigenesis. We demonstrate that the development of cSCC depends on Ly6C^hi^ monocytes, whereas LCs and macrophages are dispensable.

## Results and discussion

### Ly6C^hi^ monocytes accumulate in cSCC

**Figure 1:**
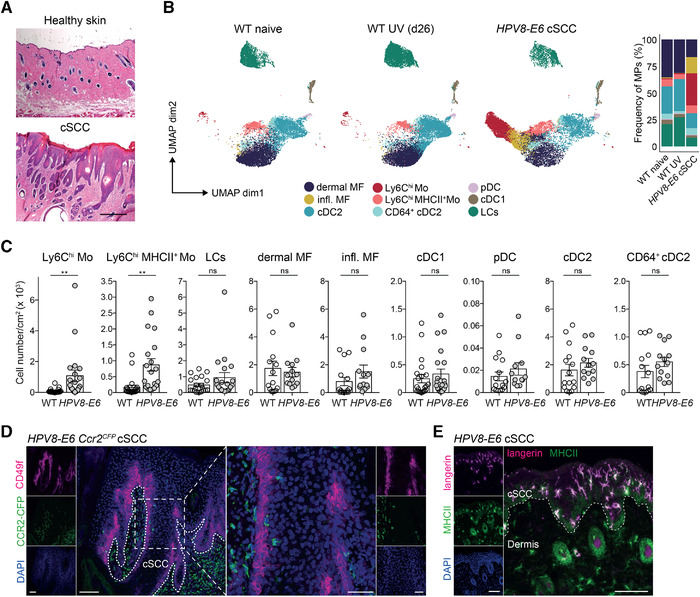
Ly6C^hi^ monocytes accumulate in cSCC. (A) H&E staining of cSCC and healthy control skin of *HPV8‐E6* mice. Scale bar: 200 μm. Representative images from three mice. (B and C) Flow cytometry analysis of mononuclear phagocytes in naïve skin of WT mice, UV‐treated skin of WT mice, or UV‐induced cSCC in *HPV8‐E6* mice. (B) Uniform manifold approximation and projection (UMAP) plots and frequency of mononuclear phagocytes (MPs) (pregated on CD45^+^CD3^−^Siglec‐F^−^Ly6G^−^) (26 dpUV). Combined data from three to five mice in each group, shown is one representative of three independent experiments. Refers to Supporting Information Figure S1A. (C) Total number of Ly6C^hi^ monocytes (Mo) (MHCII^+^ and MHCII^−^), LCs, dermal macrophages (MF), inflammatory (infl.) MF, cDC1s, plasmacytoid DCs (pDCs), cDC2s, and CD64^+^ cDC2s per cm^2^ of UV‐exposed WT skin (*n* = 15–21) or cSCC from *HPV8‐E6* mice (*n* = 13–20). Representative gating strategy shown in Supporting Fig. S1B. Data pooled from three to five individual experiments. **p < 0.01, Student's *t*‐test, two‐tailed. (D) Immunohistochemistry of CFP (CCR2, green) and CD49f (keratinocytes, magenta) in cSCC on day 28 after UVR of *HPV8‐E6 Ccr2^CFP^* mice. Representative images from three mice. Dotted line marks the border of the cSCC. Scale bar left is 100 μm, scale bar in zoomed image is 50 μm. (E) Immunohistochemistry of langerin (magenta) and MHCII (green) in UV‐induced cSCC from *HPV8‐E6* on day 42 after UVR, counterstained with DAPI (blue). Scale bar: 100 μm. Dotted line represents cSCC border. Representative image from three mice.

To phenotypically characterize the mononuclear phagocyte landscape in cSCC by flow cytometry, we exposed *HPV8‐E6* mice to a single dose of UVR, which induces acute skin inflammation within 1–3 days followed by formation of cSCC 21–28 days later (Fig. 1A) [8]. In both the control skin and in cSCC, we identified several mononuclear phagocyte populations including macrophages, Ly6C^hi^ (MHCII^+^ and MHCII^−^) monocytes, LCs, plasmacytoid DCs, and conventional DCs (cDCs). cDCs were classified into cDC1s, cDC2s, and a small frequency of cDC2s expressing CD64 (Fig. 1B, Supporting Information Fig. S1A and B), which resemble inflammatory cDC2s or monocyte‐derived cells [9]. CD64^+^ cDC2s also expressed higher levels of F4/80, CX3CR1, and CD88 than cDC2s. Naïve *HPV8‐E6* and WT skin and WT skin previously exposed to UV were comparable in immune cell composition and numbers (Fig. 1B, Supporting Information Fig. S1A, data not shown). In contrast, in cSCC the most pronounced change within the mononuclear phagocyte compartment occurred for Ly6C^hi^ (MHCII^+^ and MHCII^−^) monocytes, which accumulated in high numbers in cSCC compared to control skin (Fig. 1B and C). Macrophages were separated into a resident dermal macrophage population and an "inflammatory" macrophage population, which increased in frequency in cSCC and was characterized by higher expression of CD88, CX3CR1, and CD64 (Fig. 1B, Supporting Information Fig. S1A). Apart from monocytes, most other mononuclear phagocyte cell numbers including LCs slightly but not significantly increased in cSCC compared to control skin (Fig. 1B and C).

To assess the spatial distribution of the abundant Ly6C^hi^ (CCR2^+^) monocytes in cSCC, we performed immunohistochemistry using *HPV8‐E6 Ccr2^CFP^* reporter mice. The majority of classical monocytes (CFP^+^) localized in the dermis adjacent to the cSCC while only few CFP^+^ cells were observed within the hyperplastic area (Fig. 1D). LCs (Langerin^+^MHCII^+^) on the other hand resided in the immediate vicinity of transformed keratinocytes (Fig. 1E). Taken together, Ly6C^hi^ monocytes colonize cSCC at high numbers and mostly localize adjacent to the tumor area.

### LCs are not critical for the development of cSCC

**Figure 2:**
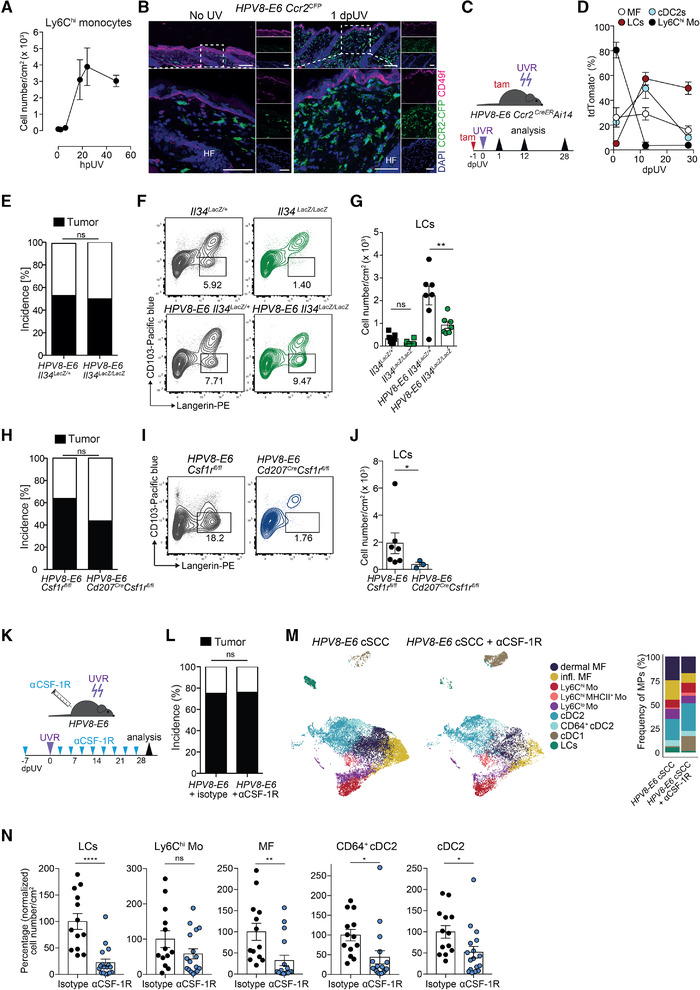
LCs are dispensable for UV‐induced cSCC development. (A) Total cell number of Ly6C^hi^ monocytes (CD45^+^Siglec‐F^−^Ly6G^−^Ly6C^hi^CD11b^+^) per cm^2^ of skin ± SEM at 0, 2, 6, 18, 24, and 48 h after UVR in *HPV8‐E6* mice, analyzed by flow cytometry. *n* = 2–5 per time point. Pooled data from two independent experiments. (B) Immunofluorescence staining of naïve skin or on day 1 after UVR in *Ccr2* ^CFP^ mice showing CFP (green), CD49f (magenta), and DAPI (blue). Big image shows enlargement of outlined region and small images show single stains. Representative images from three mice per time point. HF, hair follicle. Scale bar in upper images: 100 μm and in lower images: 50 μm. (C and D) *HPV8‐E6 Ccr2^CreER^R26‐tdTomato* (*Ai14*) mice were treated with a single dose of tamoxifen one day prior to UVR and UV‐exposed skin was analyzed by flow cytometry on days 1, 12, and 28 after UVR. Frequency of tdTomato^+^ cells among LCs, Ly6C^hi^ monocytes (Mo), macrophages (MF), and cDC2s. Representative flow cytometry plots are shown in Supporting Information Fig. S2A. Pooled data from six experiments, two independent experiments per time point, *n* = 4 per time point. (E) Percent incidence of UV‐induced cSCC development in *HPV8‐E6 Il34^LacZ/+^* (*n* = 13) and *HPV8‐E6 Il34^LacZ/LacZ^* (*n* = 14) mice. Pooled data from three individual experiments. ns, nonsignificant, Fisher's exact test. (F and G) Flow cytometry analysis of LCs (Langerin^+^CD103^−^, gated on CD45^+^Siglec‐F^−^Ly6G^−^Ly6C^−^CD64^−^MHCII^+^CD11c^+^) in skin from *Il34^LacZ/+^* (*n* = 6) and *Il34^LacZ/LacZ^* (*n* = 6) mice and cSCC from *HPV8‐E6 Il34^LacZ/+^* (*n* = 7) and *HPV8‐E6 Il34^LacZ/LacZ^* (*n* = 7) mice on day 42 after UVR. Representative FACS plots (F) and total cell numbers (G) of LCs per cm^2^ of skin (only mice that developed cSCC in the *HPV8‐E6* groups were included). Pooled data from two independent experiments, ns: non‐significant, **p < 0.01, one‐way ANOVA with Sidak's multiple comparisons test. (H) Percent incidence of UV‐induced cSCC in *HPV8‐E6 Csf1r^fl/fl^* (*n* = 11) and *HPV8‐E6 Cd207^Cre^Csf1r^fl/fl^* (*n* = 13) mice. ns: nonsignificant, Fisher's exact test. Pooled data from three independent experiments. (I and J) FACS analysis of LCs (gated on CD45^+^Siglec‐F^−^Ly6G^−^Ly6C^−^CD64^−^MHCII^+^CD11c^+^ cells) in cSCC in *HPV8‐E6 Csf1r^fl/fl^* (*n* = 6) and *HPV8‐E6 Cd207^Cre^Csf1r^fl/fl^* mice (*n* = 3). (I) Representative FACS plots and total cell number (J) of LCs per cm^2^ of skin. Data pooled from two independent experiments. *p < 0.05, Mann–Whitney test, two‐tailed. (K) Schematic representation of treatment of *HPV8‐E6* mice with αCSF‐1R antibodies and induction of cSCC (K–N). (L) Percent incidence of UV‐induced cSCC in *HPV8‐E6* control mice (*n* = 16, of which five were treated with isotype and 11 were untreated) or *HPV8‐E6* mice treated with αCSF‐1R (*n* = 25). Pooled data from four experiments. ns: nonsignificant, Fisher's exact test. (M and N) Flow cytometry analysis of mononuclear phagocytes in cSCC of *HPV8‐E6* mice treated with αCSF‐1R or *HPV8‐E6* control mice (untreated or isotype control). (M) UMAP plot and frequency from three to four mice per group and (N) percentage change in cell numbers per cm^2^ skin (normalized to cell numbers in the control group) of LCs, Ly6C^hi^ monocytes (Mo), macrophages (MF), cDC2 and CD64^+^ cDC2, as shown in the gating strategy in Supporting Information Fig. S1B. *N* = 13 for the control group (untreated and isotype‐treated combined) and *n* = 17 for the αCSF‐1R‐treated group. Pooled data from four experiments. ns: nonsignificant, **p < 0.05, Mann–Whitney test, two‐tailed.

Within hours after UVR, Ly6C^hi^ monocytes accumulated in the skin and their numbers peaked at 24 h (Fig. 2A). At this time point, they mostly resided in the dermal tissue close to the epidermis as assessed in *HPV8‐E6 Ccr2^CFP^* mice (Fig. 2B). To address whether these invading monocytes give rise to cSCC‐associated LCs or macrophages, we used *HPV8‐E6 Ccr2^CreER^Ai14* mice to fate‐map CCR2^+^ cells. A single dose of tamoxifen was administered 16 h prior to UVR to irreversibly label monocytes and trace their progeny (Fig. 2C). Shortly after UVR, 80% of monocytes were tdTomato^+^ but most had lost the label 12 days later, in accordance with their short half‐life (Fig. 2D, Supporting Information Fig. S2A). In contrast, 50% of LCs were tdTomato^+^ at day 12 and in established cSCC. Some macrophages and cDC2s appeared to be labeled directly upon tamoxifen administration (d1) likely due to their low expression of CCR2. The percentage of tdTomato^+^ macrophages and cDC2s slightly increased to 30% and 50% at 12 dpUV but decreased again to 15% and 10%, respectively, at 28 dpUV (cSCC). These data indicate that monocytes infiltrating the skin upon UVR contribute to the pool of tumor‐associated LCs and to a lesser extent, macrophages. Whether monocyte‐derived LCs have the capacity of self‐renewal in cSCC over longer periods of time or whether they will eventually be replaced remains to be shown.

To investigate whether LCs are implicated in the formation of *HPV8‐E6*‐driven cSCC, we used *HPV8‐E6 Il34^LacZ/LacZ^* mice. In these mice, LCs are absent due to the lack of IL‐34, which signals through the CSF 1 receptor (CSF‐1R) and is required for the development and maintenance of LCs [10, 11]. *HPV8‐E6 Il34^LacZ/LacZ^* mice developed cSCC with a similar incidence as control mice (Fig. 2E). However, despite the lack of IL‐34, LCs were enriched in cSCC (Fig. 2F and G), likely due to inflammation‐induced CSF‐1 expression, which can promote the differentiation of monocytes into LCs in inflammatory conditions [10, 12, 13].

To address the role of these monocyte‐derived LCs for the formation of cSCC, we used *CD207* ^Cre^ *Csf1r^fl/fl^* mice in which CSF‐1‐ and IL‐34‐mediated CSF‐1R signaling is abrogated due to the deletion of *Csf1r* in langerin‐expressing cells. This results in the absence of embryonically as well as monocyte‐derived LCs [10]. Despite the strong and specific reduction of LCs in *HPV8‐E6 CD207* ^Cre^ *Csf1r^fl/fl^* mice, the incidence of cSCC in *HPV8‐E6* *CD207* ^Cre^ *Csf1r^fl/fl^* and *HPV8‐E6 Csf1r^fl/fl^* littermate controls was comparable (Fig. 2H–J), strongly arguing against a role of LCs in tumor formation.

To further corroborate these findings, we depleted LCs in *HPV8‐E6* mice with a monoclonal anti‐CSF‐1R antibody throughout the course of cSCC development (Fig. 2K). The incidence of UVR‐induced cSCC of anti‐CSF‐1R‐treated, isotype‐treated, or untreated *HPV8‐E6* mice was similar (Fig. 2L). Of note, in addition to LCs, macrophages, which are also dependent on CSF‐1R signaling, cDC2s and CD64^+^ cDC2s were also significantly reduced by anti‐CSF‐1R treatment, while classical monocytes remained numerically and phenotypically unaffected (Fig. 2M and N, Supporting Information Fig. S2B), as previously shown [14, 15]. Altogether, these data indicate that in the *HPV8‐E6* model neither LCs nor macrophages play a significant role in the induction of cSCC.

### UVR–recruited monocytes acquire a pro‐inflammatory phenotype

**Figure 3:**
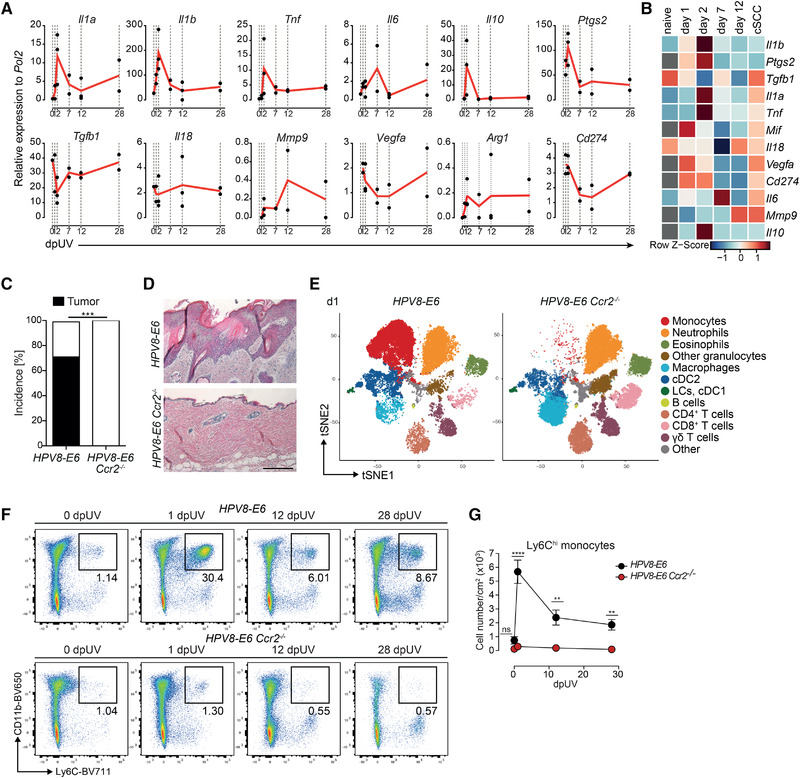
Inflammatory monocytes are critical for the development of cSCC. (A) Relative mRNA amounts of *Il1a*, *Il1b*, *Tnf*, *Il6*, *Il10*, *Ptgs2*, *Tgfb1*, *Il18*, *Mmp9*, *Vegfa*, *Arg1*, and *Cd274* measured by qPCR normalized to *Pol2* expression in sorted monocytes from naïve or UV‐irradiated *HPV8‐E6* mice on days 1, 2, 7, 12, and 28 (cSCC) after UVR. Day 1, 7, 12, 28: *n* = 2, each pooled from 2 mice; day 2: *n* = 3, each pooled from two mice; naive (day 0): *n* = 1, pooled from two mice; no data for naïve *Ptgs2, Mif, Vegfa, Cd274, Mmp9, Il10*. (B) Heatmap of data in (A), scaled for each gene; grey: data not available. (C) Percent incidence of cSCC development in *HPV8‐E6* (*n* = 35) and *HPV8‐E6 Ccr2^−/−^* (*n* = 22) mice. ***p < 0.001, Fisher's exact test. (D) Representative H&E staining of cSCC (*HPV8‐E6* mice) and skin (*HPV8‐E6 Ccr2^−/−^* mice) on day 42 after UVR. Scale bar: 200 μm. (E) t‐Distributed stochastic neighbor embedding (tSNE) plot of CD45^+^ cells on day 1 after UVR in *HPV8‐E6* and *HPV8‐E6 Ccr2^−/−^* mice. N = 2 per genotype. (F and G) Flow cytometry analysis of monocytes (Ly6C^+^CD11b^+^) among CD45^+^Siglec‐F^−^Ly6G^−^ cells in skin of naïve *HPV8‐E6* or *HPV8‐E6 Ccr2^−/‐^* mice or on day 1, 12, or 28 after UVR. Representative plots (F) and total cell numbers (G) of monocytes per cm^2^ of skin. Pooled data from three independent experiments. *n* = 5–11 mice per time point per group. Refers to Supporting Information Fig. S3A–C.

To further characterize monocytes, we sorted Ly6C^hi^ monocytes by flow cytometry from UVR‐exposed skin of *HPV8‐E6* mice at different time points to investigate the expression of genes involved in tissue inflammation and promoting a pro‐tumorigenic environment. Expression of the pro‐inflammatory cytokines *Tnf*, *Il1b*, and *Il1a* was highly increased in skin‐invading monocytes on days 1 and 2 after UVR, in accordance with the rapid inflammatory response following UVR‐exposure (Fig. 3A and B). Notably, consistent with these gene expression analyses, the majority of monocytes produced TNF‐α and/or pro‐IL‐1β in the first 2 days after UVR exposure, as measured by flow cytometry (Supporting Information Fig. S2C). On day 12, the number of monocytes expressing these cytokines was reduced to almost control levels but was substantially increased again in established cSCC. *Tgfb1* expression was low after UVR but was restored to baseline levels in cSCC, whereas *Il10* was elevated on day 2 after UVR and decreased thereafter (Fig. 3A and B).

Prostaglandins, and in particular PGE2, promote UV‐induced immunosuppression and proliferation of malignant keratinocytes [16]. We found that *Ptgs2* (the gene encoding PGE2) was highly upregulated in monocytes in the first 2 days after UVR (Fig. 3A and B). The expression of *Il6*, *Il18*, as well as macrophage‐derived factors involved in angiogenesis and ECM degradation—*Vegfa* and *Mmp9*, respectively—was generally low. *Cd274* (encoding PD‐L1) decreased immediately after UVR but increased again in monocytes derived from cSCC‐bearing mice (day 28), whereas *Pdcd1lg2* (encoding PD‐L2) was not detectable. *Arg1*, which is often enriched in immunosuppressive macrophages, was barely expressed (Fig. 3A).

Altogether, these data suggest that following UVR, monocytes rapidly infiltrate the inflamed skin and induce a pro‐inflammatory transcriptional program. This initial wave of infiltrating monocytes might create an inflammatory milieu promoting mutagenesis and transformation or could also inhibit apoptosis of damaged, pre‐malignant keratinocytes inducing their proliferation, similar to a previously described function of LCs [7].

### Mice lacking circulating monocytes are resistant to UV‐induced cSCC

To address whether Ly6C^hi^ monocytes are directly involved in UV‐induced carcinogenesis, we crossed *Ccr2* ^−/−^ mice, which lack circulating Ly6C^hi^ monocytes, to *HPV8‐E6* mice. We found no overt difference in acute inflammation upon UVR between *HPV8‐E6* and *HPV8‐E6 Ccr2^−/−^* mice, as observed by erythema formation and skin thickening (data not shown) and as assessed by total skin concentrations of IL‐1β and TNF‐α and local reactive oxygen species (ROS) production (Supporting Information Fig. S2D–F). The latter has also been implicated with mutagenesis and malignant transformation [17]. However, only *HPV8‐E6* mice developed cSCC while *HPV8‐E6 Ccr2^−/−^* mice recovered completely from UV‐induced skin inflammation and did not display any macroscopic or histologic signs of epidermal hyperplasia or dysplasia (Fig. 3C and D). Expectedly, Ly6C^hi^ monocytes were absent from the skin of UV‐irradiated *HPV8‐E6 Ccr2^−/−^* mice, while LCs and cDC1s followed similar kinetics in *HPV8‐E6* and *HPV8‐E6 Ccr2^−^^/−^* mice (Fig. 3E–G, Supporting Information Fig. S3A–C). Numbers of CD64^+^ cDC2s and macrophages were decreased in *HPV8‐E6 Ccr2^−/−^* mice, suggesting that they differentiate from infiltrating monocytes (Supporting Information Fig. S3B–C) [9]. Notably, these myeloid cell subsets were also decreased in anti‐CSF1‐R treated mice as in *HPV8‐E6 Ccr2^−/−^* mice except for Ly6C^hi^ monocytes, which were present in anti‐CSF1‐R treated mice. Yet, the former group was susceptible to the development of cSCC while *HPV8‐E6 Ccr2^−/−^* mice were resistant, suggesting a critical role for monocytes in this inflammation‐induced skin carcinogenesis.

A recent study demonstrated that IL‐33‐responsive myeloid cells are important in promoting progression of experimental SCC [18]. Whether IL‐33 signaling is also involved in the recruitment and maintenance of monocytes in our model or which other growth factors control monocyte accumulation remains to be shown.

We also observed that neutrophil cell numbers were elevated in *HPV8‐E6 Ccr2^−/‐^* mice after UVR (Supporting Information Fig. S3A–C), in agreement with previous studies *in Ccr2* ^−/−^ mice [19]. This was however not the cause for the protection from developing cSCC, since depletion of neutrophils with a monoclonal antibody against Ly6G did not affect the incidence of cSCC (Supporting Information Fig. S3D–F).

Altogether, these results indicate that the development of UVR‐induced cSCC depends on classical monocytes, which invade the skin early after UVR and are present at high numbers throughout the development of cSCC.

## Concluding remarks

Our data demonstrate that while LCs reside in the immediate vicinity of transformed keratinocytes, they are not a prerequisite for the development of cSCC in a transgenic UV‐induced model. Conversely, *HPV8‐E6* mice lacking inflammatory monocytes are completely protected from the development of cSCC. Ly6C^hi^ monocytes are recruited early to the UV‐inflamed skin, accumulate throughout tumor progression, and presumably orchestrate tumor‐promoting inflammation. The precise underlying mechanisms, however, need to be investigated further to understand how monocytes facilitate tumor growth and to identify potential druggable targets.

## Materials and methods

### Mice

All mouse strains were kept in‐house in individually ventilated cages under specific pathogen‐free conditions. *IL34^LacZ/LacZ^* and *HPV8‐E6* mice were bred in‐house [8, 10]. *Csf1r^fl/fl^* mice were kindly provided by Jeffrey Pollard [20] and *Cd207^Cre^* mice [21] were kindly provided by Björn Clausen and were kept heterozygous. *Ai14* mice were purchased from Jackson [22]. *Ccr2^CreERmKate^* (*Ccr2^CreER^*) mice were generated by Taconic Artemis [23]. *Ccr2^−/−^* mice were purchased from Taconic Biosciences [24]. *Ccr2^DTR‐CFP^* (*Ccr2^CFP^*) mice were previously described [25]. All mice were kept on a C57Bl/6 background. All described animal procedures were approved by the Swiss Veterinary Office. Both female and male mice used were between 10 and 16 weeks old.

### Induction of UV‐induced cSCC

To induce cSCC, mice were anesthetized with 6.5 mg/kg body weight xylazine (Xylasol; Graeub) and 65 mg/kg body weight ketamine (Ketasol‐100; Graeub) administered i.p. The back of the mouse was shaved. Mice were covered with aluminum foil to limit the exposed skin area to 2 × 2 cm and placed on a UV lamp (Waldmann) equipped with PUVA lamps (UVA: 320–400 nm) and UV21 lamps (UVB: 280–360 nm with a peak at about 314 nm) and exposed to a dose of 10 J/cm^2^ UVA and 1 J/cm^2^ UVB as previously described [8]. Epidermal dysplasia and cSCC started developing in susceptible strains approximately 21 days after UV radiation. Tumor incidence was generally assessed between 28 and 35 dpUV exposure.

### Blocking of CSF‐1R and Ly6G

Hybridoma cells (AFS98) were grown in Gibco Protein‐Free Hybridoma Medium II (PFHM‐II, Thermo Fisher Scientific) in CELLine™ disposable bioreactor flask (Corning, Fisher Scientific) following the manufacturer's instructions [26]. Monoclonal antibodies were purified from hybridoma supernatant using PD‐10 desalting columns following the manufacturer's protocol (GE Healthcare). Isotype control antibodies (rat IgG2a, clone 2A3) were purchased from Bio X Cell. For blocking of CSF‐1R, mice were injected with an initial dose of 2 mg followed by 0.5 mg i.p. twice per week with anti‐CSF‐1R antibody or the respective isotype control (clone 2A3). For blocking Ly6G, mice were injected i.p. 4 times with 200 μg of anti‐Ly6G (clone 1A8) (Bio X Cell) or the isotype control (rat IgG2a) every second day.

### Tamoxifen treatment

Tamoxifen solution was prepared by dissolving tamoxifen powder (Sigma) in corn oil (Sigma) to a concentration of 25 mg/mL. Five milligram of tamoxifen in 200 μL was administered by oral gavage.

### Preparation of single‐cell suspension for FACS analysis

For isolation of leukocytes mouse back skin or cSCC was floated epidermal side down on 2.4 mg/mL Dispase (Roche) in HBSS for 1.5 h at 37°C. The skin was then cut into small pieces and incubated in 0.4 mg/mL Collagenase IV (Sigma) and 0.04 mg/mL DNAse I (Sigma) in HBSS (Gibco) and 10% FBS (Gibco) for 1.5 h at 37°C. After digestion, skin suspension was homogenized with an 18G needle and syringe, filtered through a 100 μm cell strainer, washed, and the whole pellet was used for staining with antibodies against surface antigens. Single‐cell suspensions were incubated with Zombie Aqua or Zombie NIR fixable viability dye (BioLegend) and fluorochrome‐conjugated monoclonal antibodies against mouse CD45 (30‐F11), CD3 (17A2), CD4 (GK1.5), CD11b (M1/70), CD11c (N418), CD24 (M1/69), CD64 (X54‐5/7.1), CD88 (20/70), CD90 (30‐H12), CD103 (2E7), CD117 (2B8), CX3CR1 (SA011F11), Ly6C (HK1.4 or AL‐21), Ly6G (1A8), NK1.1 (PK136), MHCII I‐A/I‐E (M5/114.15.2), TCRgd (GL3), FceRIa (36951), and XCR1 (ZET) from BioLegend; CD8a (53‐6.7), CD19 (1D3), CD45 (30‐F11), CD45R (RA3‐6B2), and Siglec‐F (E50‐2440) from BD Biosciences; CD19 (1D3), CD172a (P84), MerTK (DS5MMER), and CD317 (eBio927) from ThermoFisher Scientific; and F4/80 (CI:A3‐1) from BioRad. Secondary staining was performed with fluorescently labeled streptavidin (BioLegend or BD Biosciences).

For intracellular antigen staining (i.e., langerin), cells were fixed and permeabilized for 10–20 min with BD Cytofix/Cytoperm solution (BD) and then stained with APC‐ or PE‐labeled anti‐langerin (clone 4C7, BioLegend or clone eBioRMUL.2; Thermo Fisher Scientific) antibody overnight.

For myeloid cell cytokine staining, cell suspensions were incubated in RPMI‐1640 medium containing 10 % FCS, penicillin, and streptomycin for 4 h at 37°C in the presence of BD GolgiPlug Protein transport inhibitor containing Brefeldin A (BD) at the dilution recommended by the supplier. After washing, cells were fixed and permeabilized for 30 min with BD Cytofix/Cytoperm solution (BD) and incubated overnight with antibodies against mouse IL‐1a (ALF‐161; BioLegend), IL‐1b pro‐form (NJTEN3; ThermoFisher Scientific), IL‐6 (MP5‐20F3; BioLegend), IL‐10 (JES5‐16E3; BioLegend), and TNF‐a (MP6‐XT22; BioLegend). Data were acquired on BD LSRFortessa, BD FACSymphony (BD), SP6800 (Sony Biotechnologies), or Cytek Aurora. Fluorescence‐activated cell sorting (FACS) was performed on BD FACS Aria II equipped with a 100 μm nozzle. For quantification of total cell numbers, the measured cell number was normalized to the area of skin used.

### Quantitative real‐time PCR

RNA from sorted cells was isolated using RNeasy Plus Micro isolation kit (Qiagen) following the manufacturer's instructions. Complementary DNA (cDNA) was generated using M‐MLV reverse transcriptase (ThermoFisher Scientific) in the presence of RNAse inhibitor RNAseOUT (ThermoFisher Scientific). Semi‐quantitative PCR reactions were performed using iTaq Universal SYBR Green Supermix (Bio‐Rad) and 100 nM each of specified forward and reverse primers. Custom primers for *Arg1* (forward 5′ (Fwd) – CTGGGAATCTGCATGGGCAA, reverse 5′ (Rev) – GTCTACGTCTCGCAAGCCAA), *Cd274* (Fwd – TGTGGAGAAATGTGGCGTTG, Rev – TGCCAATCGACGATCAGAGG), *Il1a* (Fwd – TGTTGCTGAAGGAGTTGCCAGA; Rev – TCTGGAAGTCTGTCATAGAGGGCA), *Il1b* (Fwd – GATCCACACTCTCCAGCTGCA, Rev – CAACCAACAAGTGATATTCTCCATG), *Il18* (Fwd – TGTGGTTCCATGCTTTCTGGAC, Rev – AGGTTTGAGGCGGCTTTCTT), *Il10* (Fwd – GGTTGCCAAGCCTTATCGGA, Rev – ACCTGCTCCACTGCCTTGCT), *Il6* (Fwd – ATGGATGCTACCAAACTGGAT, Rev – TGAAGGACTCTGGCTTTGTCT), *Mmp9* (Fwd – CGTCGTTGATCCCCACTTACT, Rev – AACACACAGGGTTTTGCCTTC), *Nos2* (Fwd – TGTGCTGTTCTCAGCCCAAC, Rev – GCAGCTTGTCCAGGGATTCT), *Pdcd1lg2* (Fwd – CTGTGCTGGGTGCTGATATTG, Rev – GGGGCTGTCACGGTGAATAA), *Ptgs2* (Fwd – TGGGCCATGGAGTGGACTTA, Rev – GGGGATACACCTCTCCACCA), *Tgfb1* (Fwd – TGACGTCACTGGAGTTGTACGG, Rev – GGTTCATGTCATGGATGGTGC), *Tnf* (Fwd – CATCTTCTCAAAATTCGAGTGACAA, Rev – TGGGAGTAGACAAGGTACAACCC), *Vegfa* (Fwd – GCGGGCTGCCTCGCAGTC, Rev – TCACCGCCTTGGCTTGTCAC) were obtained from Thermo Fisher Scientific.

### Tissue preparation and immunohistochemistry

For langerin immunostaining, skin was collected and immediately frozen in OCT cryo‐embedding medium without fixation. Tissue sections of 10 μm were cut on a Zeiss Hyrax C60 cryostat and were dried for 2 h at room temperature before fixation in acetone pre‐cooled to −20°C for 10 min, followed by two washes with neutral pH PBS.

For all other immunohistochemical staining, skin was first fixed in 4% PFA (Morphisto) for 18–24 h at 4°C, then incubated in 30% sucrose solution for 24–48 h at 4°C, embedded in OCT, and frozen. Tissue sections of 10–14 μm were dried for 2 h at room temperature and then washed in PBS.

Before staining, sections were blocked in PBS containing 10% normal goat serum (Thermo Fisher Scientific) and 1% Triton X‐100 (Sigma) for 1 h at room temperature. Sections were stained overnight at 4°C with primary antibodies against langerin (clone 4C7, APC‐conjugates; BioLegend), MHCII I‐A/I‐E (clone M5/114.15.2, FITC‐conjugated; BioLegend), GFP (used to detect also CFP, rat IgG2a, clone GF090R; Nacalai Tesque), and CD49f (clone GoH3, APC‐conjugated; BioLegend). When staining with anti‐GFP antibody, a secondary staining with goat anti‐rat IgG (Alexa Fluor 488; ThermoFisher Scientific) diluted in 5% normal goat serum (Thermo Fisher Scientific) and 0.25% Triton X‐100 (Sigma) was performed. After washing, sections were mounted in Immunoselect Antifading Mounting Medium containing DAPI (Dianova) and cover slips were sealed with nail polish.

For H&E staining, tissues were fixed in HOPE‐I reagent (DCS) for 24–72 h at 4°C, followed by dehydration for 2 h in ice‐cold HOPE‐II solution (DCS) and 6 h in ice‐cold acetone (Sigma). Dehydrated tissues were soaked in melted paraffin overnight then embedded in paraffin. Sections (2 μm) were cut on a rotary microtome (Thermo Fisher Scientific) and dried at 37°C overnight. On the next day, tissues were deparaffinized in 2 changes of xylol (10 min each) and rehydrated sequentially in 90% ethanol (6 min), 80% ethanol (6 min), 70% ethanol (6 min), and distilled H_2_O. Deparaffinized sections were stained for 5 min in Harris hematoxylin (Sigma), then washed for 5 min in running tap water, and counterstained with eosin (Medite) for 1 min. Sections were then dehydrated sequentially in 95% ethanol (2 × 3 min), 100% ethanol (2 × 3 min), and xylol (2 × 5 min). After drying cover slips were mounted using Eukitt quick‐hardening mounting medium (Sigma).

### Microscopy and image analysis

Immunofluorescence images were acquired on Leica SP5 confocal microscope. Brightfield microscopy images were acquired on Olympus BX41 microscope. Images were analyzed using ImageJ software.

### In vivo ROS detection

Mice were injected i.p. with 25 mg/kg body weight L‐012 (Wako) dissolved in water and anesthetized with isoflurane. After 20 min, luminescence was measured with the IVIS Lumina S5 (PerkinElmer). Average radiance (p/s/cm^2^/sr) was calculated for the UVR‐exposed area in UVR‐treated mice or for an equal area in naïve mice.

### Tissue lysis and ELISA

Mouse skin was homogenized in ELISA lysis buffer (50 mM pH 7.4 Tris (Sigma), 5 mM EDTA, 150 mM NaCl (Sigma), 1% NP‐40 complemented with EDTA‐free protease inhibitor cocktail (Roche), and phosphatase inhibitor cocktail (Roche)) with 1.4 mm zirconium oxide beads (Precellys) in a FastPrep tissue homogenizer (MP Biomedicals). After 15‐min incubation on ice, tissue lysates were sonicated for 30 s and centrifuged for 15 min at 4°C at maximum speed in a table‐top centrifuge to remove cell debris. Total protein content from the supernatant was measured by BCA assay kit (ThermoFisher Scientific) following the manufacturer's instructions. ELISA for IL‐1b (kit by Biolegend) and TNF‐a (kit by Thermo Fisher) were performed according to the manufacturer's instructions. Signal was measured in triplicates using a microplate reader (Tecan). Calculated concentrations of the cytokines were then normalized per total protein content for each sample.

### Data analysis

Flow cytometry was conducted in line with published guidelines [27]. FlowJo X was used to analyze flow cytometry data. Computational analysis of high‐dimensional flow cytometry data, including t‐distributed stochastic neighbor embedding, uniform manifold approximation and projection dimensionality reduction, FlowSOM clustering, and visualization of marker expression, was performed in R as previously described [28]. In brief, samples were pre‐gated (on live CD45^+^CD3^−^Ly6G^−^SiglecF^−^) in FlowJo, exported as individual fcs files and imported in R where they were merged in one data frame. Merged data was transformed with the arcsinh function and normalized to fit values between 0 and 1. t‐Distributed stochastic neighbor embedding, uniform manifold approximation and projection, and FlowSOM algorithms were applied on the combined normalized data. For plotting of overlaid marker expression, overlaid clusters and cluster frequency, the ggplot2 package was used. For heatmap visualization of flow cytometry data, median marker expression was calculated with R and visualized using the heatmap.2 function. For heatmap visualization of RT‐PCR data, mean marker expression was calculated with R and visualized using the heatmap.2 function.

Statistical analysis and visualization of cell numbers, tumor incidence, and gene expression were performed using the GraphPad Prism.

## Conflict of interest

The authors declare no commercial or financial conflict of interest.

### Peer review

The peer review history for this article is available at https://publons.com/publon/10.1002/eji.202048841

**Abbreviations**
cDCs: conventional DCs
CSF‐1R: CSF 1 receptor
cSCC: cutaneous squamous cell carcinoma
LCs: Langerhans cells
UVR: UV radiation

## Supporting information

Supporting Information

## Data Availability

Data that support the findings of this study are available from the corresponding author upon request.
