# Supplementary material for: Monocytes promote UV‐induced epidermal carcinogenesis
Source: Eur J Immunol. 2021 Apr 2;51(7):1799–808. doi: 10.1002/eji.202048841 (PMC8359952; doi:10.1002/eji.202048841)
Supplement: Supplementary file 1 — Supporting Information [file EJI-51-1799-s001.pdf]

Supporting Information Figure S1

A

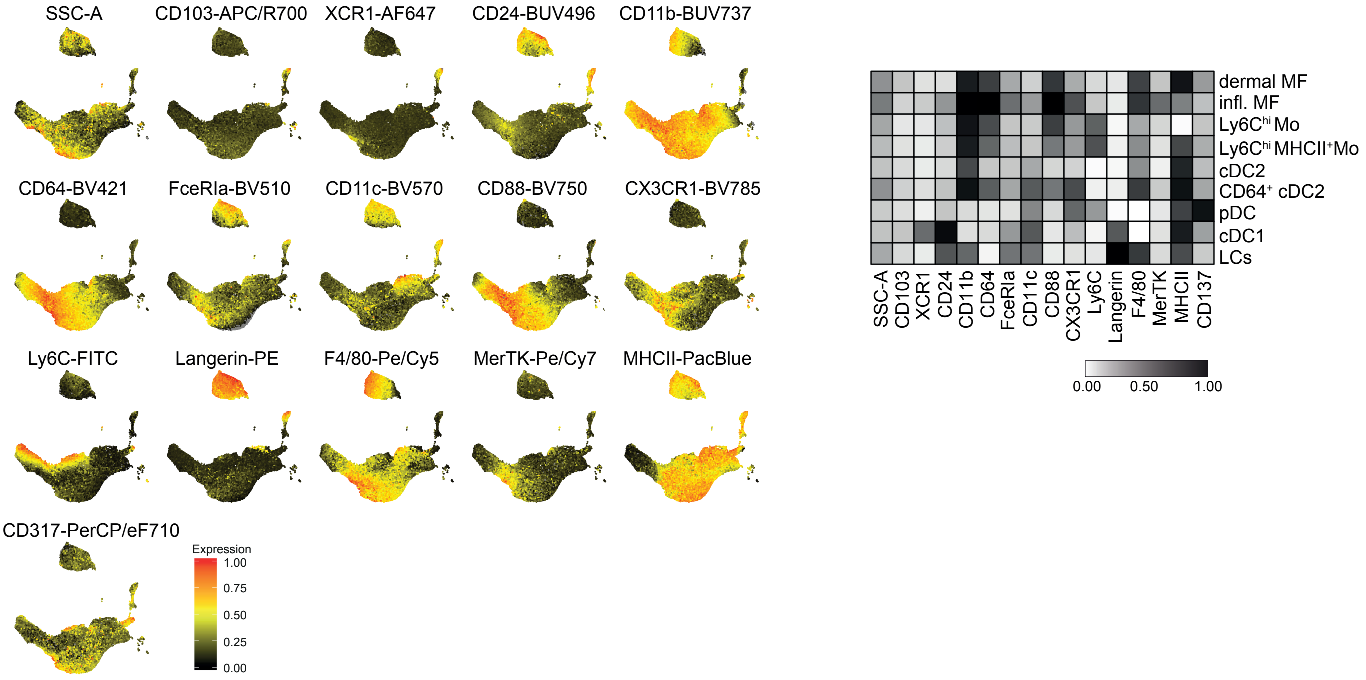

B

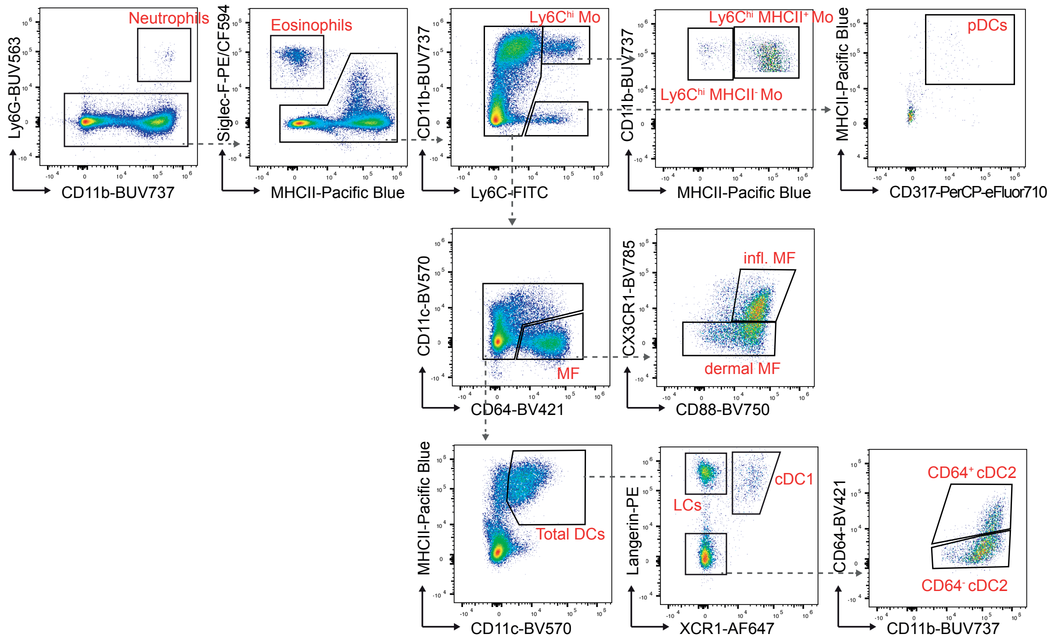

Supporting Information Figure S1. Mononuclear phagocytes in cSCC. Refers to Figure 1. (A) FACS analysis of mononuclear phagocytes (pregated on $CD45^{+}CD3^{-}$Siglec-$F^{-}Ly6G^{-}$) in naïve or UV-treated skin of WT mice and UV-induced cSCC in *HPV8-E6* mice. Normalized expression of markers used to identify the different subsets in Fig. 1B overlaid on the combined UMAP plot and median expression in identified subsets are shown in the heatmap (right). n = 3-5 mice per group. (B) Representative gating strategy for the identification of $Ly6C^{hi}$ ($MHCII^{+}$ and $MHCII^{-}$) monocytes (Mo), dermal macrophages (dermal MF), inflammatory (infl.) MF, LCs, pDCs, cDC1s and cDC2s ($CD64^{+}$ and $CD64^{-}$) in the skin. Refers to Figures 1-3.

Supporting Information Figure S2

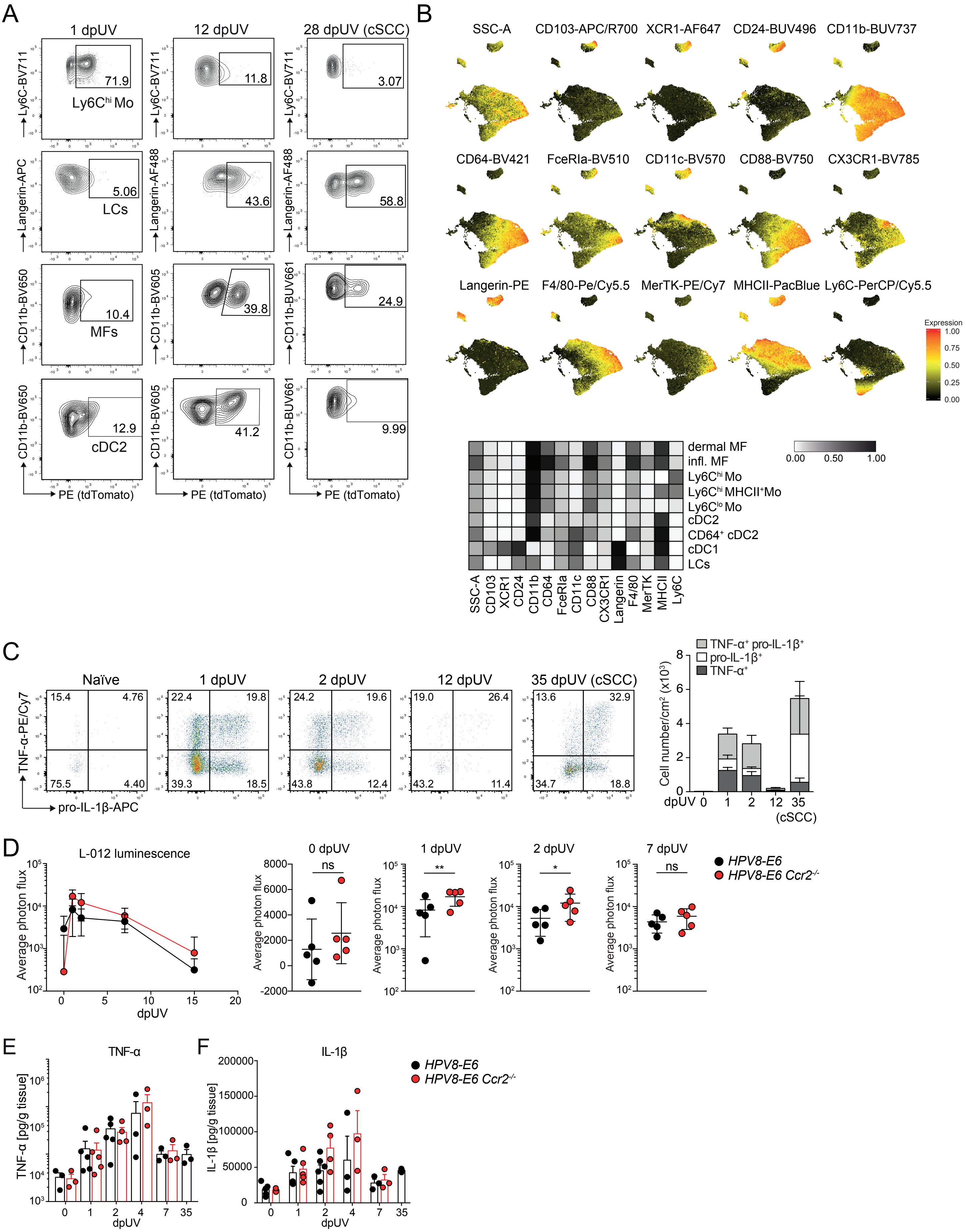

Supporting Information Figure S2. Cytokine expression in the skin after UVR. Refers to Figure 2 and 3. (A) *HPV8-E6 Ccr2^{CreER}R26-tdTomato* (*Ai14*) mice were treated with a single dose of tamoxifen one day prior to UVR and UV-exposed skin was analyzed by flow cytometry on days 1, 12 and 28 after UVR. Representative FACS plots are shown for the frequency of tdTomato^{+} cells among LCs, Ly6C^{hi} monocytes (Mo), cDC2s and macrophages (MF). Pooled data from 6 experiments, 2 independent experiments per time point, n = 4 per time point. Refers to Fig. 2C-D. (B) Normalized expression of markers used to identify the different subsets shown in Fig. 2M overlaid on the combined UMAP plot and median expression in identified subsets are shown in the heatmap. (C) FACS analysis of intracellular expression of TNF-α and pro-IL-1β in Ly6C^{hi}CD11b^{+} monocytes (pregated on CD45^{+}Siglec-F^{-}Ly6G^{-}) in the skin of *HPV8-E6* mice at indicated time points after UVR and in cSCC (day 35 after UVR). Shown are representative FACS plots and total cell numbers per cm^2 skin of TNF-α^{+}, pro-IL-1β^{+} and TNF-α^{+}pro-IL-1β^{+} monocytes. Day 0, 2, 12: n = 5 per time point; day 1 and 35: n = 3 per time point. Pooled data of 3 independent experiments. (D) *In vivo* ROS and RNS in skin measured by luminescence (average photon counts per second per cm^2 +/- SEM) after L-012 injection at day 0, 1, 2, 7 and 15 after UVR. n = 5 mice per group. ** $p < 0.01$, * $p < 0.05$, ns = non-significant, two-way ANOVA with Tukey correction for multiple comparisons. (E-F) Concentrations of TNF-α (E) and IL-1β (F) in skin in *HPV8-E6* and *HPV8-E6 Ccr2$^{-/-}$* mice on day 0, 1, 2, 4, 7 after UVR and in cSCC in *HPV8-E6* mice (day 35 after UVR). N = 3 per time point.

Supporting Information Figure S3

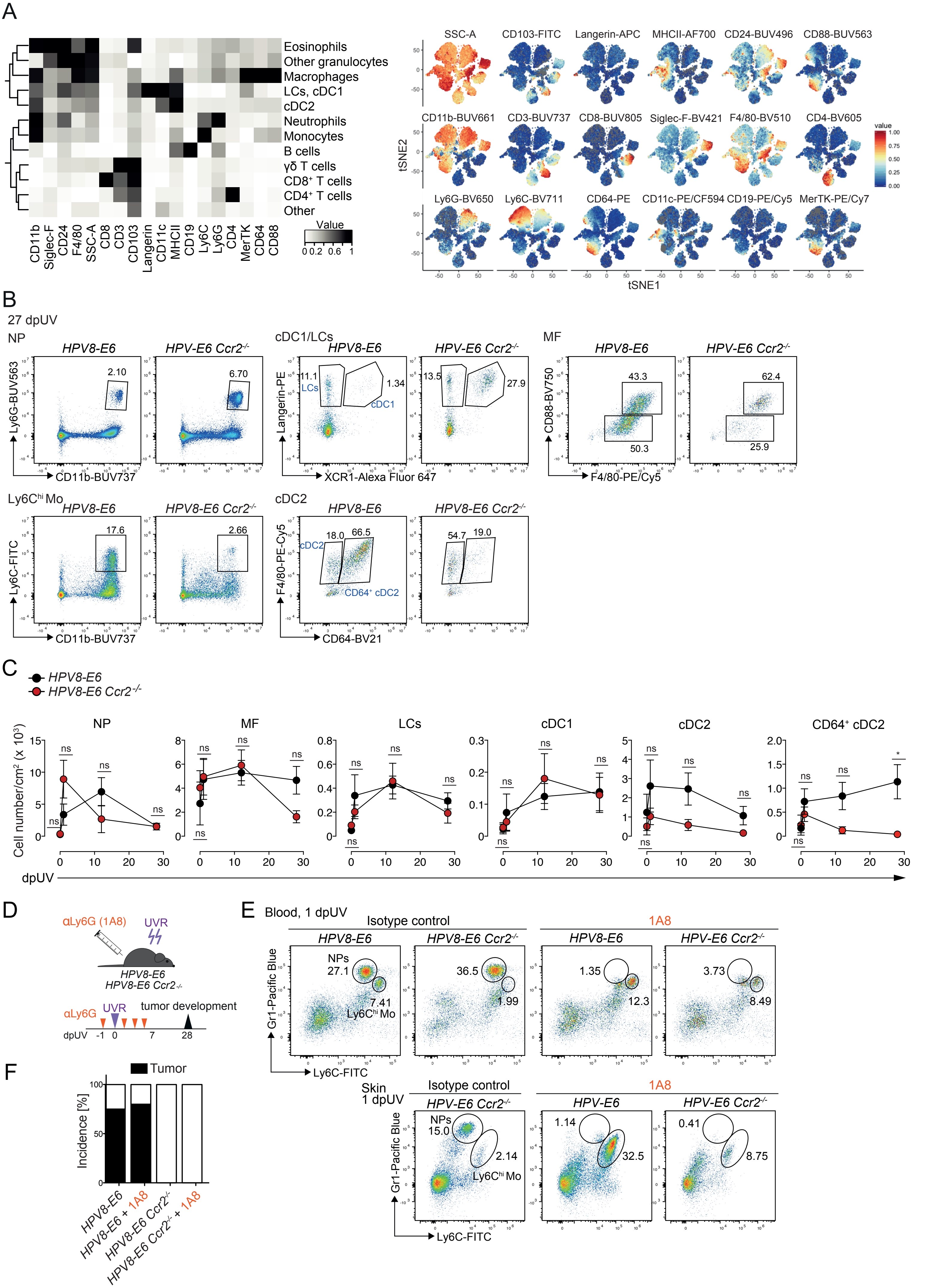

Supporting Information Figure S3. Immune cells in *HPV8-E6* and *HPV8-E6 Ccr2*$^{-/-}$ mice after UVR. Refers to Figure 3. (A) Heatmap and overlaid tSNE plots representing normalized expression of markers used to identify lymphoid and myeloid cell populations in Figure 3E. (B-C) Representative FACS plots (27 dpUV) (B) and cell numbers (C) of neutrophils (NPs), macrophages (MFs), LCs, cDC1s and cDC2s per cm^2 of skin from *HPV8-E6* or *HPV8-E6 Ccr2*$^{-/-}$ mice on day 0, 1, 12 and 27/28 after UV irradiation (C) (pregated as shown in Supporting Information Fig. S1B). Pooled data from 3-4 independent experiments. n = 5-11 mice per time point per group. Two-way ANOVA with Sidak's multiple comparisons test. (D) Schematic representation of treatment with αLy6G antibody (1A8) for depletion of neutrophils (E-F). (E) Representative FACS plots showing neutrophils (Gr1$^+$Ly6C^{lo}) and monocytes (Gr1^{lo}Ly6C^{hi}) on day 1 after UVR (day 2 after first injection of αLy6G antibody or isotype control) in blood and skin. N = 2. (F) Incidence of cSCC in *HPV8-E6* and *HPV8-E6 Ccr2*$^{-/-}$ mice treated with αLy6G antibody (1A8) or isotype control. n = 4 for *HPV8-E6* (isotype), n = 5 for *HPV8-E6* (1A8) and *HPV8-E6 Ccr2*$^{-/-}$ (isotype, 1A8).
